# Real-World Data of Different Immune Checkpoint Inhibitors for Non-Small Cell Lung Cancer in China

**DOI:** 10.3389/fonc.2022.859938

**Published:** 2022-03-15

**Authors:** Kang Miao, Xiaotong Zhang, Hanping Wang, Xiaoyan Si, Jun Ni, Wei Zhong, Jing Zhao, Yan Xu, Minjiang Chen, Ruili Pan, Mengzhao Wang, Li Zhang

**Affiliations:** Department of Pulmonary and Critical Care Medicine, Peking Union Medical Hospital, Chinese Academy of Medical Science and Peking Union Medical College, Beijing, China

**Keywords:** immune checkpoint inhibitors (ICIs), immunotherapy, non-small cell lung cancer (NSCLC), objective response rate (ORR), progression-free survival (PFS), real-world data (RWD)

## Abstract

**Background:**

Patients treated with immunotherapy in the real-world may have significantly different responses to those meeting inclusion criteria for random controlled clinical studies. There is a partial overlap in approved indications for the use of the different immune checkpoint inhibitors (ICIs) currently available. A comprehensive assessment of the efficacy, safety and economic effects of various ICIs is a problem that clinicians need to address.

**Methods:**

Analyzed real-world data was collected from non-small cell lung cancer (NSCLC) patients who were treated with ICIs from hospitalized patients in the Lung Cancer Center of Peking Union Medical College Hospital between 2018 and 2021. The objectives were to evaluate the efficacy and safety of different ICIs for the treatment of NSCLC in China and to investigate the factors affecting their curative effects.

**Results:**

Overall, 351 patients were included in the retrospective study. The median PFS for the NSCLC patient cohort treated with medication regimens that included ICIs was 9.5 months, with an ORR of 47.3%. There were no significant discrepancies in efficacy and safety between the different ICIs administered. Factors that had the greatest impact on the efficacy of ICIs were the disease stage, ECOG-PS scores and treatment lines. Gender, age, smoking history, PD-L1 TPS expression, history of targeted therapy and irAEs all had a degree of influence on patient prognosis.

**Conclusions:**

The study reports the experience of real-world usage of ICIs for the treatment of NSCLC patients in China. The results were generally consistent with those of clinical trials, while the efficacy and safety of different ICIs exhibited no statistically significant differences. Therefore, physicians can make a comprehensive choice based on the indications and cost of different ICIs and the preferences of patients.

## Background

Immune checkpoint inhibitors (ICIs) are a class of antitumor drug that activate lymphocytes, which then attack tumor cells by relieving immune checkpoint-mediated immune-suppression (1, 2). In 2015, two types of ICIs (nivolumab and pembrolizumab) were approved by the U.S. Food and Drug Administration for the treatment of patients with advanced non-small cell lung cancer (NSCLC) (3). Subsequently, immunotherapy was officially introduced into China with the launch of nivolumab and pembrolizumab in June 2018 and July 2018, respectively (4).

Clinical studies under strictly controlled experimental conditions provided an objective assessment of the efficacy and safety of various ICIs, and were the main basis for their approval. However, in the real-world, patients given immunotherapy may have significantly different treatment criteria from those enrolled in clinical trials (5). Based on Keynote 407 and Keynote 189 studies, pembrolizumab was approved for the treatment of NSCLC as combination chemotherapy (6, 7). Further approval of pembrolizumab for monotherapy in PD-L1 positive NSCLC was based on Keynote 042 and Keynote 024 studies (8, 9). Pembrolizumab, which has the most extensive clinical study data available, is usually the first-choice treatment for patients with NSCLC. However, there is partial overlap in the approved indications for different ICIs. Currently, 8 ICIs have been approved for clinical use in China, including 2 imported PD-1 inhibitors (nivolumab and pembrolizumab), 2 imported PD-L1 inhibitors (atezolizumab and durvalumab) and 4 PD-1 inhibitors manufactured in China (camrelizumab, tislelizumab, sintilimab and toripalimab) (10). Therefore, a comprehensive assessment of the efficacy, safety and economic effects of various ICIs has become a problem that clinicians need to address. Studies based on real-world data (RWD) provide a reference for understanding patient outcomes outside of clinical trials and better guide treatment decisions (11). Most of the current clinical studies used patients on conventional chemotherapy as the controls, but unfortunately this resulted in a paucity of data with regard to direct efficacy comparisons between the different ICIs administered. An RWD-based cross-sectional study will help to compare the efficacy and safety of different ICIs.

Thus, a real-world retrospective observational study was conducted to explore the efficacy and safety of different types of ICIs in a cross-sectional manner. In addition, we also investigated the possible risk factors that may affect the efficacy of ICIs to provide guidance for optimization of clinical decision making.

## Methods

### Study Objectives

This was a retrospective, observational, single-center study that collected and analyzed data from real-world NSCLC patients treated with ICIs. The objectives were to evaluate the efficacy and safety of different ICIs for the treatment of NSCLC in China and to investigate the impact of factors affecting their curative effects.

### Ethics Approval Statement

The study was performed in accordance with the principles of the Declaration of Helsinki and approved by the Ethics Review Committee of Peking Union Medical College Hospital (2020-12-24/HS-2195). Written informed consent was provided by all enrolled patients.

### Data Collection

Data from the digital hospital information management system (HIS database) of Peking Union Medical College Hospital were analyzed. Inclusion criteria were patients with NSCLC who had been treated with ICIs and followed up for at least 6 months.

Information about each enrolled patient was carefully documented including: gender; age; pathology type; disease stage; date of diagnosis; targeted drug history; history of smoking; alcohol consumption; Eastern Cooperative Oncology Group performance status (ECOG-PS) scores; PD-L1 TPS (PD-L1 tumor cell proportion score: The percentage of PD-L1 membrane stained tumor cells in the total number of tumor cells); type of ICIs administered; time of initiation of ICI therapy; lines of ICIs; combination chemotherapy; combination anti-vascular therapy; real-world PFS; optimal efficacy; and immune-related adverse effects (irAEs).

### Statistical Analysis

Descriptive analyses of the characteristics of patients, their disease states and treatment were conducted who were then stratified into subgroups of interest. Enumeration data are described according to statistical quantities and percentages. Only one data measurement was included (age in years), presented as the median and interquartile range, because the data were not normally distributed. The implications of different characteristics for PFS were compared using the Kaplan-Meier method and a log-rank test. Median survival estimates and the hazard ratio (HR) from a Cox proportional hazards model, as well as the 95% confidence intervals (CIs) are reported. The PFS was defined as the duration from the onset of treatment with ICIs to disease progression or death, which was assessed based on the date of disease progression as indicated by CT or PET/CT scans confirmed by the supervising physician, or in-hospital death recorded in the HIS database. Logistic regression was used to analyze the effects of different factors on the ORR, and the ORR for each subgroup of interest, the odds ratios (OR) from logistic regression and the corresponding 95% CIs are reported. Indicators from univariate analysis that reached the threshold (*P* < 0.1) were employed in the multivariate analysis, with a *P*-value < 0.05 deemed to be a statistically significant finding. Fisher exact test was used to analyze differences in the incidence rate of irAEs between various ICIs. All statistical analyses were conducted using SPSS (ver. 24.0) and Kaplan-Meier survival curves were constructed using GraphPad Prism (ver. 8.0.1).

## Results

Data from 1,832 patients who received therapy from January 2018 to December 2021 were analyzed. Among them, 461 were NSCLC patients treated with ICIs. To ensure completeness of the survival information, only data from patients followed up for ≥ 6 months were included in the analysis. A total of 110 patients were excluded due to insufficient follow-up, and finally 351 patients were formally included in the study.

**Figure 1:**
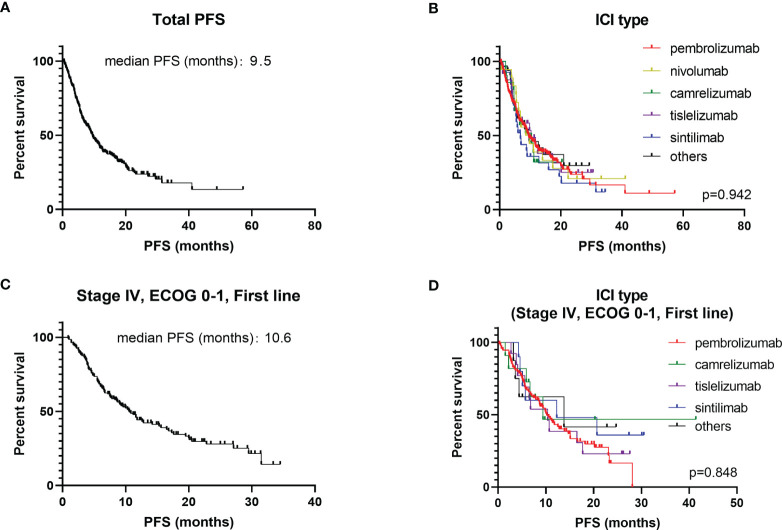
Kaplan-Meier plot of PFS for different ICIs. **(A)** PFS for all NSCLC patients; **(B)** stratified according to different ICIs (all NSCLC patients); **(C)** PFS for stage IV, first line, ECOG 0 – 1 patients; **(D)** stratified according to different ICIs (stage IV, first line, ECOG 0 – 1 patients).

The majority (73.8%) were male, with a median age of 65 years. Slightly more patients had non-squamous carcinoma (55.0%) than squamous carcinoma (40.2%), with the remaining 17 patients not otherwise specified (NOS). About 64.7% patients had a history of smoking and 32.8% a history of alcohol consumption. The majority of NSCLC patients were able to take care of themselves at the time of starting immunotherapy (ECOG-PS scores 0 – 1), but the other 11.7% required long-term bed rest (ECOG-PS scores 2 – 4). Circa 78.3% patients treated with ICIs were at stage IV and 19.4% patients with the EGFR/ALK mutation had previously received targeted therapy. The type of ICIs used for NSCLC was predominantly pembrolizumab (65.2%), with the remaining therapy including nivolumab (5.1%), camrelizumab (6.0%), tislelizumab (6.8%) and sintilimab (7.7%). Basic information had no significant impact on the choice of different types of ICIs except for histology pathology type (*P* = 0.015) (**Table S1**). ICIs were given as first-line treatment to 53.6% patients, 28.8% for second line treatment and 9.1% for third-line treatment and beyond. ICIs were administered to 30 patients for other reasons including maintenance treatment after radiotherapy and for neoadjuvant treatment before surgery. Immunotherapy was usually administered in combination with chemotherapy (75.2%) for 4–6 courses before being switched to maintenance therapy of ICIs. Only 10.5% patients chose immunotherapy in conjunction with anti-vascular therapy (**Table 1**). The regimen containing ICIs produced an overall median PFS of 9.5 months, with an ORR of 47.3% (**Figure 1A**). The 1-year PFS rate was 35.9% and the 2-year PFS rate 8.5%. There was no significant difference between the efficacy of various ICIs, with good overlap of survival curves (*P* = 0.942) (**Figure 1B** **)**. The median PFS for pembrolizumab was 9.6 months with an ORR of 45.0%; for nivolumab, the median PFS was 9.2 months with an ORR of 50%; for camrelizumab, the median PFS was 10.4 months with an ORR of 47.6%; for tislelizumab, the median PFS was 10.3 months with an ORR of 54.2%; and for sintilimab, the median PFS was 6.8 months, with an ORR of 51.9% (**Table 2**). The longest median PFS was produced by camrelizumab (10.4 months) and the highest ORR by tislelizumab (54.2%).

**Table 1:** Basic information.

Basic information	Number	Percentage
**Patients**	351	100.0%
**Sex**		
Male	259	73.8%
Female	92	26.2%
**Age**	65 (60-70)	
< 60	97	27.6%
60-74	221	63.0%
≥ 75	33	9.4%
**Histology**		
Non-squamous carcinoma	193	55.0%
Squamous carcinoma	141	40.2%
NOS	17	4.8%
**Lung cancer stage**		
III	76	21.7%
IV	275	78.3%
**History of targeted therapy**		
No	283	80.6%
Yes	68	19.4%
**ICI type**		
Pembrolizumab	229	65.2%
Nivolumab	18	5.1%
Camrelizumab	21	6.0%
Tislelizumab	24	6.8%
Sintilimab	27	7.7%
Others	32	9.1%
**Line of therapy**		
First-line	188	53.6%
Second-line	101	28.8%
Third-line and beyond	32	9.1%
Others	30	8.5%
**Combined chemotherapy**		
No	87	24.8%
Mono chemotherapy	41	11.7%
Doublet chemotherapy	223	63.5%
**Combined anti-vascular therapy**		
No	314	89.5%
Yes	37	10.5%
**Smoking status**		
No	124	35.3%
Yes	227	64.7%
**Drinking status**		
No	236	67.2%
Yes	115	32.8%
**ECOG-PS**		
0	148	42.2%
1	162	46.2%
2	27	7.7%
3	7	2.0%
4	7	2.0%
**Recorded irAEs (need glucocorticoids)**	60	
Grade 1	8	13.3%
Grade 2	18	30.0%
Grade 3	14	23.3%
Grade 4	20	33.3%
**PD-L1 TPS**	91	
0%	21	23.1%
1-49%	37	40.7%
≥ 50%	33	36.3%
**Primary efficacy assessment**		
PR	166	47.3%
SD	130	37.0%
PD	55	15.7%

NOS, not otherwise specified; ICI, immune checkpoint inhibitor; ECOG-PS, Eastern Cooperative Oncology Group performance status; irAEs, immune-related adverse events; TPS, tumor cell proportion score; PR, partial response; SD, stable disease; PD, progressive disease.

**Table 2:** Differences in efficacy between ICIs (all NSCLC patients).

		Cox regression				Logistic regression			
	number	Median PFS	HR	95%CI	P-value	ORR	OR	95%CI	P-value
**Total**	351								
**Pembrolizumab**	229	9.6 months	Reference		0.943	45.0%	Reference		0.894
**Nivolumab**	18	9.2 months	0.945	0.545-1.638	0.840	50.0%	0.817	0.313-2.135	0.681
**Camrelizumab**	21	10.4 months	1.077	0.610-1.901	0.799	47.6%	0.899	0.367-2.201	0.816
**Tislelizumab**	24	10.3 months	0.917	0.547-1.537	0.741	54.2%	0.692	0.297-1.609	0.392
**Sintilimab**	27	6.8 months	1.212	0.773-1.903	0.402	51.9%	0.759	0.342-1.687	0.499
**Others**	32	9.7 months	0.909	0.563-1.466	0.695	53.1%	0.721	0.344-1.514	0.388

ICIs, immune checkpoint inhibitors; PFS, progression-free survival; HR, hazard ratio; 95% CI, 95% confidence interval; ORR, objective response rate; OR, odds ratio;

Different application mode of ICIs and patient baseline characteristics may have an impact on prognosis. Lung cancer stage, line of therapy and ECOG-PS scores were the three factors that have the greatest impact on survival (**Table S2**). Thus, the population of stage IV NSCLC patients with ECOG-PS scores of 0 – 1, given ICIs as first-line treatment were selected as the subgroup study population (**Table 3**). The median PFS for this subgroup population was 10.6 months, with an ORR of 51.5% (**Figure 1C**). The most commonly administered ICIs were pembrolizumab, camrelizumab, tislelizumab and sintilimab. Nivolumab is less commonly used in first-line therapy due to its price and lack of associated single-agent indications. The results of survival analysis showed that there was no significant difference in efficacy between the various ICIs in this subgroup, with a median PFS of 9.4–13.8 months and ORR of 47.9–63.6% (*P* = 0.846) (**Figure 1D**).

**Table 3:** Differences in efficacy between ICIs (Stage IV, first line, ECOG 0-1 patients).

		cox regression				logistic regression			
	number	Median PFS	HR	95%CI	p-value	ORR	OR	95%CI	p-value
**Total**	136								
**Pembrolizumab**	94	10.2 months	Reference		0.811	47.9%	Reference		0.768
**Camrelizumab**	11	9.4 months	0.779	0.313-1.940	0.592	63.6%	0.525	0.144-1.913	0.329
**Tislelizumab**	13	10.4 months	0.825	0.409-1.662	0.590	53.8%	0.787	0.246-2.519	0.687
**Sintilimab**	10	12.3 months	0.664	0.285-1.550	0.344	60.0%	0.612	0.162-2.311	0.469
**Others**	8	13.8 months	0.703	0.256-1.993	0.495	62.5%	0.551	0.125-2.439	0.432

ICIs, immune checkpoint inhibitors; PFS, progression-free survival; HR, hazard ratio; 95% CI, 95% confidence interval; ORR, objective response rate; OR, odds ratio.

**Figure 2:**
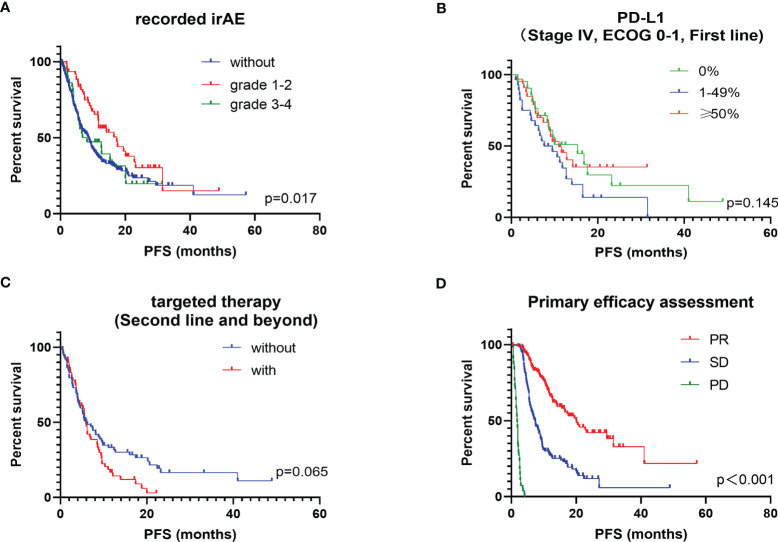
Kaplan-Meier plot of PFS for subgroups of interest. **(A)** stratified according to recorded irAEs; **(B)** stratified according to PD-L1; **(C)** stratified according to targeted therapy history in post-line patients; **(D)** stratified according to primary efficacy assessment.

For the safety analysis, immune-related adverse effects (irAEs) that required glucocorticoids intervention or discontinuation of ICIs were counted. A total of 60 patients (17.1%) had concerning irAEs, of which 34 (9.7%) were of grade 3 or higher. The types of adverse events mainly included immune-related pneumonia (39 cases, 11.1%), liver toxicity (8 cases, 2.2%), skin toxicity (13 cases, 3.7%), colitis (10 cases, 2.8%), myocarditis (4 cases, 1.1%), and other types of irAEs (9 cases, 2.6%) (**Table 4**). There was no statistical difference in the incidence of irAEs or severe irAEs for the different ICIs administered (*P* = 0.607). Death occurred in 9 patients due to severe irAEs, including 5 cases of pneumonia, 2 cases of myocarditis, 1 case of colitis and 1 case of liver toxicity. Additionally, patients who developed grade 1 to 2 irAEs (median PFS: 17.4 months, ORR: 61.7%) had a significantly better survival prognosis than those without irAEs (median PFS: 8.7 months, ORR: 44.3%) and those patients who developed grades 3 to 4 irAEs (median PFS: 7.5 months, ORR. 44.4%) (*P* = 0.017) (**Figure 2A**).

**Table 4:** Differences in safety between ICIs.

ICI		Pembrolizumab	Camrelizumab	Tislelizumab	Nivolumab	Sintilimab	Others	Total
Patients		229	21	24	18	27	32	351
**Pneumonia**	**All grade**	26 (11.4%)	2 (9.6%)	3 (12.5%)	2 (11.2%)	2 (7.4%)	4 (12.6%)	39 (11.1%)
	**≥3 grade**	10 (4.4%)	1 (4.8%)	2 (8.3%)	1 (5.6%)	1 (3.7%)	2 (6.3%)	22 (4.8%)
**Myocarditis**	**All grade**	2 (0.9%)	1 (4.7%)	1 (4.2%)	0 (0.0%)	0 (0.0%)	0 (0.0%)	4 (1.1%)
	**≥3 grade**	1 (0.4%)	1 (4.7%)	1 (4.2%)	0 (0.0%)	0 (0.0%)	0 (0.0%)	3 (0.8%)
**Skin toxicity**	**All grade**	9 (3.9%)	0 (0.0%)	1 (4.2%)	1 (5.6%)	1 (3.1%)	1 (3.7%)	13 (3.7%)
	**≥3 grade**	4 (1.7%)	0 (0.0%)	1 (4.2%)	0 (0.0%)	1 (3.1%)	1 (3.7%)	7 (2.0%)
**Colitis**	**All grade**	7 (3.1%)	0 (0.0%)	1 (4.2%)	0 (0.0%)	2 (7.4%)	0 (0.0%)	10 (2.8%)
	**≥3 grade**	4 (1.7%)	0 (0.0%)	1 (4.2%)	0 (0.0%)	2 (7.4%)	0 (0.0%)	7 (2.0%)
**Liver toxicity**	**All grade**	6 (2.6%)	0 (0.0%)	1 (4.2%)	0 (0.0%)	0 (0.0%)	1 (3.1%)	8 (2.2%)
	**≥3 grade**	4 (1.7%)	0 (0.0%)	0 (0.0%)	0 (0.0%)	0 (0.0%)	0 (0.0%)	4 (1.1%)
**Others**	**All grade**	4 (1.7%)	1 (4.7%)	0 (0.0%)	1 (5.6%)	2 (7.4%)	1 (3.1%)	9 (2.6%)
	**≥3 grade**	2 (0.9%)	0 (0.0%)	0 (0.0%)	1 (5.6%)	1 (3.1%)	0 (0.0%)	4 (1.1%)
**Total**	**All grade**	41 (17.9%)	3 (14.3%)	4 (16.7%)	3 (16.7%)	4 (14.8%)	5 (15.6%)	60 (17.1%)
	**≥3 grade**	22 (9.6%)	2 (9.6%)	3 (12.5%)	2 (11.2%)	2 (7.4%)	3 (9.4%)	34 (9.7%)

ICI, immune checkpoint inhibitor.

The patients were stratified according to their basic condition and treatment regimen. Factors included gender, age, pathology type, disease stage, type of ICIs, treatment line of ICIs, combination chemotherapy, combination anti-vascular therapy, history of smoking, history of alcohol consumption and ECOG-PS scores. After univariate Cox regression analysis, most factors were found to influence PFS to some extent (**Figures S1A–K**). Females had a significantly worse prognosis than males (*P* = 0.005) and also the prognosis was worse in patients < 60 years old and in those ≥ 75 years old (*P* = 0.085). Patients with squamous carcinoma had a slightly better survival benefit from treatment with ICIs than those with non-squamous carcinoma (*P* = 0.059). The survival benefit resulting from ICIs therapy was significantly better in NSCLC patients with stage III disease compared to those with stage IV disease (*P* < 0.001). The prolongation of PFS produced by giving ICIs as first-line treatment was longer than that for post-line treatment (*P* < 0.001). In terms of combination therapy, a platinum-based chemotherapy regimen was significantly better than mono chemotherapy or de-chemotherapy regimens (*P* < 0.001). However, instead of contributing to the prolongation of PFS, a combination with anti-vascular therapy had a negative effect (*P* = 0.041). De-chemotherapy was chosen by 46.7% of patients who adopted ICIs in combination with anti-vascular therapy, while only 9.1% patients without combined anti-vascular therapy chose the de-chemotherapy regimen (**Figure S1L** **)**. Patients with a history of smoking may benefit more from ICI therapy (*P* < 0.001). In addition, a poor general condition (ECOG-PS scores 2 to 4) was a significant associated risk factor for a poor prognosis (*P* < 0.001). Eight indicators, with significant differences in the univariate Cox regression, were included in the multivariate Cox regression analysis. It was found that the 3 indicators with the most significant influence on PFS were the disease stage, lines of ICIs administered and the ECOG-PS score (**Table S2**). A similar conclusion was reached when data were analyzed in terms of the ORR for NSCLC patients with therapeutic regimens containing ICIs. The overall ORR for the NSCLC cohort who received treatment regimens that included ICIs was 47.3%. After multifactorial logistic regression analysis, the 3 most influential risk factors for ORR remained the disease stage, line of ICIs and the ECOG-PS score (**Table S3**).

Other factors that may influence the efficacy of ICIs were also explored, mainly the PD-L1 TPS expression status, history of targeted therapy and the results of first efficacy assessment on overall PFS. PD-L1 TPS expression was recorded in 91 patients. Patients with high PD-L1 TPS expression (≥ 50%) had a longer PFS benefit compared to those with a low PD-L1 TPS expression (1–49%). Surprisingly, patients with negative PD-L1 TPS expression did not show a worse survival benefit, which was slightly better than those with low PD-L1 TPS expression (*P* = 0.145) (**Figure 2B**). Previous studies concluded that patients with positive driver genes were the population with a poor prognosis for immunotherapy (12, 13). Of the 351 patients we studied, 133 received ICIs as a backline treatment, among which 59 had received targeted therapy. In post-line therapy, patients who had received targeted therapy had an ORR of 31.1% and a median PFS of 5.9 months, while patients without driver mutations had an ORR of 28.8% and a median PFS of 6.0 months. Although the survival benefit did not reach a statistically significant difference between the two groups (*P* = 0.065), a trend of shorter PFS benefit was observed in patients with a history of targeted therapy on the survival curves (**Figure 2C**). In clinical practice, the therapeutic efficacy evaluated by impactology was generally conducted after every 2 cycles of treatment, while initial efficacy provided a good indication of the long-term survival benefit. The median PFS was 7.4 months for patients with a first evaluation of stable disease (SD) and 20.1 months for patients with a first evaluation of a partial response (PR) (*P* < 0.001) (**Figure 2D**).

## Discussion

To the best of our knowledge, this is the first large study conducted in China to cross-sectionally compare the efficacy and safety of different ICIs with RWD. The overall median PFS was 9.5 months and the ORR was 47.3%. The survival data were significantly better than those reported in earlier real-world studies, which may be attributed to a difference in the ratio of patients enrolled (14, 15). Recently, with the rapid development of immunotherapy, the use of ICIs has gradually expanded to become first-line treatment for patients with NSCLC (16). For real-world application of ICIs, Khozin et al. reported a median PFS time of 3.2 months, while Areses et al. reported a median PFS of 4.8 months (15, 16). However, ICIs were used primarily for post-line therapy in the latter study, whereas the population included in the present study was comprised of a much greater proportion of patients given first-line ICIs treatment. In contrast to real-world studies, randomized controlled trials tend to have strict screening criteria for enrolling subjects. We singled out patients with stage IV disease, ECOG-PS scores of 0 to 1 and who had received first-line ICIs treatment, which are factors considered in the screening criteria for most clinical trials. In this subgroup, the median PFS time was 10.6 months with an ORR of 51.5%, which was generally consistent with data from the clinical studies of first-line application of ICIs combined with chemotherapy (PFS: 8.0–11.3 months, ORR: 48.3–64.8%) (6, 7, 17).

The choice of ICIs has been a troubling issue for physicians, as clinical trials of different ICIs cannot be compared cross-sectionally (18). Therefore, we sought to explore the discrepancies in the efficacy and safety of different ICIs in a real-world study. Pembrolizumab in combination with chemotherapy for NSCLC demonstrated a significant survival benefit over chemotherapy in the Keynote 407 and Keynote 189 studies, with a median PFS of 8.0–9.0 months and ORR of 48.3 – 57.9% (6, 7). In our real-world cohort of patients, pembrolizumab exhibited a median PFS of 9.6 months and an ORR of 45.0%, similar to the results of clinical studies. NSCLC patients treated with camrelizumab had median PFSs of 8.5 – 11.3 months and ORRs of 60.0 – 64.8% in the Camel-sq study and Camel study, respectively (17, 19). In terms of absolute values of median PFS and ORR, the efficacy of camrelizumab seems to be better than pembrolizumab. However, in our cohort of patients, there was no significant difference between the efficacy of camrelizumab (median PFS: 10.4 months; ORR: 47.6%) and pembrolizumab. Tislelizumab produced a median PFS of 7.6 months and 9.7 months in the Ratinale 307 and Ratinale 304 studies, with ORRs of 72.5% and 57.4%, respectively (20). In our cohort of patients, it produced a median PFS of 10.3 months with an ORR of 54.2%, which was not statistical difference from pembrolizumab. In the Orient 11 study, sintilimab combined with chemotherapy resulted in a median PFS of 8.9 months and an ORR of 51.9% for non-squamous NSCLC patients (21). In our cohort, the ORR for sintilimab was 51.9%, similar to that reported in the Orient 11 study, and not significantly dissimilar to other ICIs. However, it had a median PFS of only 6.8 months, significantly lower than other ICIs in our cohort of patients. Considering the impact of baseline information on survival prognosis, we analyzed the baseline treatment information and found that the majority population treated with sintilimab were post-line. Therefore, we further analyzed a subgroup population (stage IV, first line, ECOG 0–1) and found that the efficacy of sintilimab was not any worse than that of other ICIs.

For the safety analysis, as our study was a retrospective analysis, recording the occurrence of all adverse events was rather difficult. Therefore, we focused on irAEs that required glucocorticoid interventions or suspension of ICIs, which were of most concern to clinicians. The results showed that there was no statistically difference in the incidence of irAEs produced by different PD-1 inhibitors. The most common irAEs that require glucocorticoids treatment was checkpoint inhibitor pneumonitis (CIP). The incidence of CIP in our cohort was 11.1%, and 4.8% for severe CIP (grade 3 or above), similar to previous reports (3.5–19%) (22). In addition, there was a significant correlation between the incidence of CIP and radiotherapy. CIP occurred in 26.3% patients who had chest radiotherapy during immunotherapy, compared with 8.6% patients without radiotherapy (*P* = 0.001). As there were no significant differences in efficacy and safety between the various types of ICIs, physicians can make a comprehensive choice based on indications and the cost of different ICIs, and the preference of patients. In fact, the occurrence of irAEs does not always imply a poor prognosis. Patients who developed severe irAEs need to discontinue ICIs and be treated with glucocorticoids or even immunosuppressive drugs, which can promote tumor growth (23). Therefore, severe irAEs do lead to a poor prognosis for NSCLC patients. However, mild irAEs not only do not affect tumor treatment, they have a positive impact on prognosis, compared to those who do not develop irAEs. Recently, irAEs have emerged as a potential clinical biomarker for predicting the efficacy of ICIs (24). Our results reconfirmed the idea that patients with grade 1-2 irAEs had a longer median PFS than those without irAEs.

More than 75% of patients included in the present study were over 60 years old. Previous research suggested that the efficacy of ICIs, as well as the patient tolerance of ICIs, was not significantly correlated with age (25). However, our results indicated that the 60-74-year-old population exhibited a longer PFS time, but there was no significant difference in ORR. Younger patients (< 60 years old) tend to be metabolically active with high tumor malignancy (26), while elderly patients (≥ 75 years old) frequently have poor ECOG-PS scores, which is an inherent risk factor for the efficacy of ICIs (27). The influence of gender on the efficacy of ICIs has been reported in a number of studies. Keynote 024 revealed that males were more likely to benefit from ICI therapy (8). In contrast, the real-world study by Khozin et al. found that females achieved longer PFS and OS times after ICI therapy (16). The findings of the present study showed that males benefited more than females from ICI therapy in the overall NSCLC cohort of patients.

Researchers have been trying to establish the best combination immunotherapy regimens. Currently, the most commonly used immunotherapy regimens include ICIs combined with chemotherapy, as well as single ICI (6, 9). In addition, double immunization combination regimens and the combination of ICIs with anti-vascular therapy are also directions worthy of further investigation (28, 29). Currently, CTLA-4 inhibitors have not yet been marketed in China, thus real-world data on double immunization combination regimens are lacking. In contrast, anti-vascular drugs, such as bevacizumab and anlotinib, have occupied a pivotal position for the treatment of NSCLC. The efficacy and safety of immunotherapy in combination with anti-vascular therapy has been confirmed (30). However, the findings of the present study revealed that anti-vascular therapy combined with immunotherapy failed to improve the efficacy of ICIs. On the one hand, the majority of patients who opted for combined anti-vascular therapy chose a concurrent de-chemotherapy regimen. On the other hand, a 4-drug combination regimen (platinum-based chemotherapy + ICIs + anti-vascular therapy) has not yet been authorized for relevant clinical applications (29). In the real-world, this regimen has been used primarily in young patients with high expectations of a good prognosis or having liver metastases. Anti-vascular therapy has been used in 16.7% of young patients (< 60 years old) compared to 8.6% of elderly patients (≥ 75 years old) (*P* = 0.036), and in 17.1% of patients with liver metastases compared to 7.8% patients without liver metastases. These data might partially explain why the median PFS was shorter after combination anti-vascular therapy.

Despite great heterogeneity, PD-L1 expression levels remain the best biomarker to predict the efficacy of PD-1 inhibitors in NSCLC patients (31, 32). Patients with a high PD-L1 TPS expression (> 50%) had a better PFS benefit than those with low expression of PD-L1 TPS (< 50%). However, the survival prognosis of patients with low PD-L1 TPS expression appears to be inferior to that of patients with negative PD-L1 TPS expression. This finding indicates that PD-L1 still has a great defect as a biomarker for predicting the efficacy of immunotherapy, and indirectly supports the idea that PD-L1 expression is highly heterogeneous in tumor tissues. Khozin et al. concluded that the presence of driver gene mutations was a risk factor for poor prognosis after treatment with ICIs (33). To avoid interference by treatment lines, NSCLC patients who used ICIs as first-line treatment were excluded. The majority of first-line treatment options for patients with driver mutations are targeted therapy. We divided the patient population who would receive post-line ICI therapy into 2 subgroups, namely those given chemotherapy as first-line treatment and those who received targeted therapy as first-line treatment. The results indicated that the presence of driver gene mutations and a history of targeted therapy did affect the efficacy of subsequent treatment with ICIs in the long-term PFS but not the ORR.

Inevitably, there were some limitations to our study. First, the HIS database only recorded the survival endpoints of a small number of patients (patients who died in our hospital) and thus there is a lack of OS data. Second, the gene mutation status, PD-L1 expression information and irAEs were incompletely recorded in the HIS database, which may lead to analysis bias. Third, the number of cases for each type of ICIs except pembrolizumab were small, so that the results may be affected by random effects. Fourth, our study focused on the current status of ICI therapy in China, with all enrolled patients being Asian and therefore the results may not be completely applicable to other races.

## Conclusions

A single-center, real-world study in China was conducted to explore the efficacy and safety of different ICIs for NSCLC therapy, along with the impact of patient baseline characteristics and treatment regimens on prognosis. The overall median PFS for the NSCLC patient cohort treated with regimens including ICIs was 9.5 months, with an ORR of 47.3%. There were no significant discrepancies in efficacy and safety between the different ICIs administered. Factors that had the greatest impact on the efficacy of ICIs were the disease stage, ECOG-PS scores and treatment lines. It is noteworthy that gender, age, smoking history, PD-L1 expression, history of targeted therapy, and irAEs all had a degree of influence on patient prognosis.

## Data Availability Statement

The original contributions presented in the study are included in the article/**Supplementary Material**. Further inquiries can be directed to the corresponding authors.

## Ethics Statement

The studies involving human participants were reviewed and approved by Ethics Review Committee of Peking Union Medical College Hospital. The patients/participants provided their written informed consent to participate in this study.

## Author Contributions

KM: Drafting the manuscript and revising it for important intellectual content. XZ, HW, XS, JN, WZ, JZ, YX, MC, and RP: Provision of clinical data, article revision and correction. LZ and MW: Substantial contributions to the conception or design of the work and final approval of the version to be published.

## Conflict of Interest

The authors declare that the research was conducted in the absence of any commercial or financial relationships that could be construed as a potential conflict of interest.

## Publisher’s Note

All claims expressed in this article are solely those of the authors and do not necessarily represent those of their affiliated organizations, or those of the publisher, the editors and the reviewers. Any product that may be evaluated in this article, or claim that may be made by its manufacturer, is not guaranteed or endorsed by the publisher.

## Supplementary Material

Supplementary Figure 1 Kaplan-Meier plot of PFS for subgroups for basic Information. **(A)** stratified according to sex; **(B)** stratified according to age; **(C)** stratified according to histology; **(D)** stratified according to lung cancer stage; **(E)** stratified according to the ICI type; **(F)** stratified according to the line of therapy; **(G)** stratified according to whether combined with chemotherapy; **(H)** stratified according to whether combined with anti-vascular therapy; **(I)** stratified according to smoking status; **(J)** stratified according to drinking status; **(K)** stratified according to the ECOG-PS score; **(L)** Correlation between chemotherapy and anti-vascular therapy. PFS, progression-free survival; ECOG-PS, Eastern Cooperative Oncology Group performance status; ICI, immune checkpoint inhibitor; PR: partial response; SD: stable disease; PD: progressive disease.

## Abbreviations

ICIs, immune checkpoint inhibitors; NSCLC, non-small cell lung cancer; ORR, objective response rate; PFS, progression-free survival; RWD, real-world data; ECOG-PS, Eastern Cooperative Oncology Group performance status; irAEs, immune-related adverse effects; HR, hazard ratio; CIs, confidence intervals; OR, odds ratios; NOS, not otherwise specified; CIP, checkpoint inhibitor pneumonitis.
