# Supplementary figures and images for: Real-World Data of Different Immune Checkpoint Inhibitors for Non-Small Cell Lung Cancer in China
Source: Front Oncol. 2022 Mar 15;12:859938. doi: 10.3389/fonc.2022.859938 (PMC8982065; doi:10.3389/fonc.2022.859938)

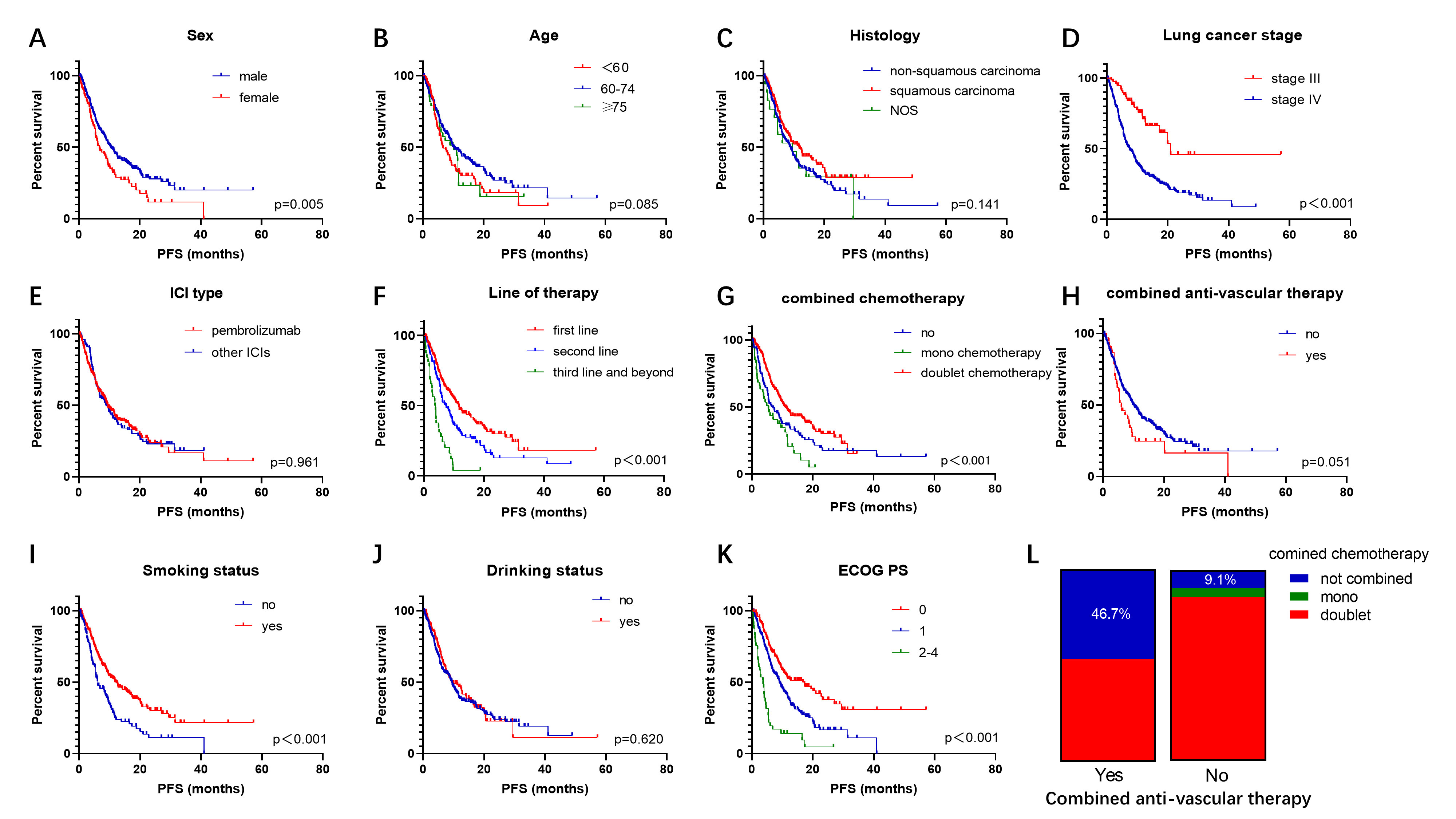

Supplement: Supplementary Figure 1 — Kaplan-Meier plot of PFS for subgroups for basic Information. (A) stratified according to sex; (B) stratified according to age; (C) stratified according to histology; (D) stratified according to lung cancer stage; (E) stratified according to the ICI type; (F) stratified according to the line of therapy; (G) stratified according to whether combined with chemotherapy; (H) stratified according to whether combined with anti-vascular therapy; (I) stratified according to smoking status; (J) stratified according to drinking status; (K) stratified according to the ECOG-PS score; (L) Correlation between chemotherapy and anti-vascular therapy. PFS, progression-free survival; ECOG-PS, Eastern Cooperative Oncology Group performance status; ICI, immune checkpoint inhibitor; PR: partial response; SD: stable disease; PD: progressive disease. [file Image_1.tif]
