# Supplementary material for: Real-World Data of Different Immune Checkpoint Inhibitors for Non-Small Cell Lung Cancer in China
Source: Front Oncol. 2022 Mar 15;12:859938. doi: 10.3389/fonc.2022.859938 (PMC8982065; doi:10.3389/fonc.2022.859938)
Supplement: Supplementary file 2 [file Table_1.docx]

**Table S1. Data of basic information between ICIs**

| **Basic information** | **Pembrolizumab** | **Camrelizumab** | **Tislelizumab** | **Nivolumab** | **Sintilimab** | **Others** | **P-value** |
| --- | --- | --- | --- | --- | --- | --- | --- |
| **Sex** |  |  |  |  |  |  | 0.148 |
| male | 166 | 18 | 17 | 10 | 24 | 24 |  |
| female | 63 | 3 | 7 | 8 | 3 | 8 |  |
| **Age** |  |  |  |  |  |  | 0.207 |
| ＜60 | 59 | 3 | 6 | 6 | 12 | 11 |  |
| ≥60 | 170 | 18 | 18 | 12 | 15 | 21 |  |
| **Histology** |  |  |  |  |  |  | 0.015 |
| non-squamous carcinoma | 128 | 14 | 9 | 15 | 9 | 18 |  |
| squamous carcinoma | 87 | 6 | 14 | 2 | 18 | 14 |  |
| NOS | 14 | 1 | 1 | 1 | 0 | 0 |  |
| **Lung cancer stage** |  |  |  |  |  |  | 0.346 |
| III | 51 | 4 | 7 | 1 | 2 | 11 |  |
| IV | 178 | 17 | 17 | 17 | 25 | 21 |  |
| **Smoking status** |  |  |  |  |  |  | 0.387 |
| no | 83 | 7 | 6 | 7 | 6 | 15 |  |
| yes | 146 | 14 | 18 | 11 | 21 | 17 |  |
| **Drinking status** |  |  |  |  |  |  | 0.555 |
| no | 154 | 15 | 14 | 15 | 16 | 22 |  |
| yes | 75 | 6 | 10 | 3 | 11 | 10 |  |
| **ECOG PS** |  |  |  |  |  |  | 0.664 |
| 0-1 | 198 | 20 | 23 | 16 | 24 | 29 |  |
| 2-4 | 31 | 1 | 1 | 2 | 3 | 3 |  |

ICIs, immune checkpoint inhibitors; NOS, not otherwise specified; ECOG-PS, Eastern Cooperative Oncology Group performance status.
