# Supplementary material for: Real-World Data of Different Immune Checkpoint Inhibitors for Non-Small Cell Lung Cancer in China
Source: Front Oncol. 2022 Mar 15;12:859938. doi: 10.3389/fonc.2022.859938 (PMC8982065; doi:10.3389/fonc.2022.859938)
Supplement: Supplementary file 3 [file Table_2.docx]

**Table S2. Cox regression for PFS**

|  |  | | **Univariate Cox regression** | | | **Multivariate Cox regression** | | | | |
| --- | --- | --- | --- | --- | --- | --- | --- | --- | --- | --- |
|  | **PFS (months)** | | **HR** | **95%CI** | **P-value** | **HR** | **95%CI** | | | **P-value** |
| **Sex** |  | |  |  | 0.005 |  |  | | | 0.331 |
| Male | 10.5 | | Reference group | | | Reference group | | | | |
| Female | 6.8 | | 1.496 | 1.1291.982 |  | 0.829 | 0.5681.210 | | |  |
| **Age** |  | |  |  | 0.085 |  |  | | | 0.262 |
| <60 | 6.7 | | Reference group | | | Reference group | | | | |
| 60-74 | 10.3 | | 0.731 | 0.5430.984 | 0.039 | 0.779 | 0.5741.058 | | | 0.110 |
| ≥75 | 10.2 | | 0.948 | 0.5911.520 | 0.825 | 0.762 | 0.4561.273 | | | 0.299 |
| **Histology** |  | |  |  | 0.141 |  |  | | |  |
| Nonsquamous carcinoma | 8.8 | | Reference group | | |  | |  |  | |
| Squamous carcinoma | 11.8 | | 0.768 | 0.5841.010 | 0.059 |  |  | | |  |
| NOS | 9.5 | | 1.057 | 0.5981.869 | 0.848 |  |  | | |  |
| **Lung cancer stage** |  | |  |  | ＜0.001 |  |  | | | 0.001 |
| III | 21.0 | | Reference group | | | Reference group | | | | |
| IV | 7.5 | | 3.100 | 2.0304.733 |  | 2.184 | 1.3533.526 | | |  |
| **ICI type** |  | |  |  | 0.961 |  |  | | |  |
| Pembrolizumab | 9.6 | | Reference group | | |  |  | | |  |
| Others | 9.0 | | 1.007 |  |  |  |  | | |  |
| **Line of therapy** |  | |  |  | ＜0.001 |  |  | | | 0.026 |
| First line | 11.6 | | Reference group | | | Reference group | | | | |
| Second line | 7.4 | | 1.532 | 1.1532.035 | 0.003 | 1.301 | 0.9361.807 | | | 0.117 |
| Third line and beyond | 3.8 | | 3.742 | 2.4645.683 | ＜0.001 | 1.972 | 1.1913.264 | | | 0.008 |
| **combined chemotherapy** |  | |  |  | ＜0.001 |  |  | | | 0.970 |
| No | 7.0 | | Reference group | | | Reference group | | | | |
| Mono chemotherapy | 5.5 | | 1.512 | 0.9912.307 | 0.055 | 1.047 | 0.6571.668 | | | 0.847 |
| Doublet chemotherapy | 11.1 | | 0.682 | 0.5060.919 | 0.012 | 0.987 | 0.6991.394 | | | 0.942 |
| **combined antivascular therapy** |  |  | |  | 0.051 |  |  | | | 0.742 |
| No | 10.0 | | Reference group | | | Reference group | | | | |
| Yes | 6.0 | | 1.474 | 0.9982.178 |  | 0.930 | 0.6061.429 | | |  |
| **Smoking status** |  | |  |  | ＜0.001 |  |  | | | 0.112 |
| No | 6.2 | | Reference group | | | Reference group | | | | |
| Yes | 12.4 | | 0.554 | 0.4250.721 |  | 0.743 | 0.5151.072 | | |  |
| **Drinking status** |  | |  |  | 0.620 |  |  | | |  |
| No | 9.5 | | Reference group | | |  |  | | |  |
| Yes | 9.8 | | 0.933 | 0.7071.230 |  |  |  | | |  |
| **ECOG PS** |  | |  |  | ＜0.001 |  |  | | | ＜0.001 |
| 0 | 16.8 | | Reference group | | | Reference group | | | | |
| 1 | 9.1 | | 1.690 | 1.2672.255 | ＜0.001 | 1.464 | 1.0811.984 | | | 0.014 |
| 24 | 3.7 | | 4.438 | 2.9766.616 | ＜0.001 | 3.540 | 2.2625.540 | | | ＜0.001 |
| **Total** | 9.5 | |  |  |  |  |  | | |  |

PFS, progression free survival; HR, hazard ratio; 95% CI, 95% confidence interval; NOS, not otherwise specified; ICI, immune checkpoint inhibitor; ECOG PS, Eastern Cooperative Oncology Group performance status.
