# Supplementary material for: Real-World Data of Different Immune Checkpoint Inhibitors for Non-Small Cell Lung Cancer in China
Source: Front Oncol. 2022 Mar 15;12:859938. doi: 10.3389/fonc.2022.859938 (PMC8982065; doi:10.3389/fonc.2022.859938)
Supplement: Supplementary file 4 [file Table_3.docx]

**Table S3. Logistic regression for ORR**

|  |  | **Univariate logistic regression** | | | **Multivariate logistic regression** | | |
| --- | --- | --- | --- | --- | --- | --- | --- |
|  | **ORR** | **OR** | **95%CI** | **p-value** | **OR** | **95%CI** | **p-value** |
| **Sex** |  |  |  | 0.394 |  |  |  |
| Male | 48.6% | Reference group | | |  |  |  |
| Female | 43.5% | 1.232 | 0.763-1.988 |  |  |  |  |
| **Age** |  |  |  | 0.724 |  |  |  |
| ＜60 | 50.5% | Reference group | | |  |  |  |
| 60-74 | 45.7% | 1.213 | 0.752-1.956 | 0.429 |  |  |  |
| ≥75 | 48.5% | 1.085 | 0.492-2.390 | 0.840 |  |  |  |
| **Histology** |  |  |  | 0.207 |  |  |  |
| Non-squamous carcinoma | 43.0% | Reference group | | |  |  |  |
| Squamous carcinoma | 52.5% | 0.683 | 0.442-1.057 | 0.087 |  |  |  |
| NOS | 52.9% | 0.671 | 0.248-1.812 | 0.431 |  |  |  |
| **Lung cancer stage** |  |  |  | ＜0.001 |  |  | 0.022 |
| III | 72.4% | Reference group | | | Reference group | | |
| IV | 40.4% | 3.870 | 2.216-6.757 |  | 2.251 | 1.125-4.504 |  |
| **ICI type** |  |  |  | 0.234 |  |  |  |
| Pembrolizumab | 45.0% | Reference group | | |  |  |  |
| Others | 51.6% | 0.766 | 0.493-1.189 |  |  |  |  |
| **Line of therapy** |  |  |  | ＜0.001 |  |  | 0.139 |
| First line | 54.8% | Reference group | | | Reference group | | |
| Second line | 35.6% | 2.188 | 1.329-3.601 | 0.002 | 1.446 | 0.823-2.541 | 0.200 |
| Third line and beyond | 12.5% | 8.482 | 2.862-25.136 | ＜0.001 | 3.510 | 1.076-11.447 | 0.037 |
| **Combined chemotherapy** |  |  |  | ＜0.001 |  |  | 0.171 |
| No | 35.6% | Reference group | | | Reference group | | |
| Mono chemotherapy | 22.0% | 1.968 | 0.833-4.651 | 0.123 | 1.300 | 0.509-3.322 | 0.583 |
| Doublet chemotherapy | 56.5% | 0.426 | 0.255-0.711 | 0.001 | 0.645 | 0.356-1.168 | 0.148 |
| **Combined anti-vascular therapy** |  |  |  | 0.027 |  |  | 0.155 |
| No | 49.4% | Reference group | | | Reference group | | |
| yes | 29.7% | 2.304 | 1.101-4.824 |  | 1.766 | 0.806-3.867 |  |
| **Smoking status** |  |  |  | 0.415 |  |  |  |
| No | 44.4% | Reference group | | |  | | |
| Yes | 48.9% | 0.833 | 0.537-1.293 |  |  |  |  |
| **Drinking status** |  |  |  | 0.318 |  |  |  |
| No | 49.2% | Reference group | | |  |  |  |
| Yes | 43.5% | 1.257 | 0.803-1.968 |  |  |  |  |
| **ECOG PS** |  |  |  | ＜0.001 |  |  | 0.023 |
| 0 | 59.5% | Reference group | | | Reference group | | |
| 1 | 43.8% | 1.880 | 1.197-2.953 | 0.006 | 1.367 | 0.838-2.229 | 0.210 |
| 2-4 | 17.1% | 7.124 | 2.963-17.126 | ＜0.001 | 3.646 | 1.429-9.300 | 0.007 |
| **Total** | 47.3% |  |  |  |  |  |  |

ORR, objective response rate; OR, odds ratio; 95% CI, 95% confidence interval; NOS, not otherwise specified; ICI, immune checkpoint inhibitor; ECOG-PS, Eastern Cooperative Oncology Group performance status.
